# Pre-motor versus motor cerebral cortex neuromodulation for chronic neuropathic pain

**DOI:** 10.1038/s41598-021-91872-2

**Published:** 2021-06-16

**Authors:** Igor Lavrov, Timur Latypov, Elvira Mukhametova, Brian N. Lundstrom, Paola Sandroni, Kendall Lee, Bryan Klassen, Matt Stead

**Affiliations:** 1 Department of Neurology, Mayo Clinic, Rochester, MN USA; 2 Department of Biomedical Engineering, Mayo Clinic, Rochester, MN USA; 3 Institute of Fundamental Medicine and Biology, Kazan Federal University, Kazan, Russia; 4 Skolkovo Institute of Science and Technology, Moscow, Russia; 5 Division of Brain, Imaging, and Behaviour Systems Neuroscience, Krembil Research Institute, Toronto Western Hospital, University Health Network, Toronto, ON Canada; 6 Institute of Medical Science, Faculty of Medicine, University of Toronto, Toronto, ON Canada; 7 Department of Neurologic Surgery, Mayo Clinic, Rochester, MN USA

**Keywords:** Neuropathic pain, Sensorimotor processing

## Abstract

Electrical stimulation of the cerebral cortex (ESCC) has been used to treat intractable neuropathic pain for nearly two decades, however, no standardized approach for this technique has been developed. In order to optimize targeting and validate the effect of ESCC before placing the permanent grid, we introduced initial assessment with trial stimulation, using a temporary grid of subdural electrodes. In this retrospective study we evaluate the role of electrode location on cerebral cortex in control of neuropathic pain and the role of trial stimulation in target-optimization for ESCC. Location of the temporary grid electrodes and location of permanent electrodes were evaluated in correlation with the long-term efficacy of ESCC. The results of this study demonstrate that the long-term effect of subdural pre-motor cortex stimulation is at least the same or higher compare to effect of subdural motor or combined pre-motor and motor cortex stimulation. These results also demonstrate that the initial trial stimulation helps to optimize permanent electrode positions in relation to the optimal functional target that is critical in cases when brain shift is expected. Proposed methodology and novel results open a new direction for development of neuromodulation techniques to control chronic neuropathic pain.

## Introduction

Chronic pain is clinically identified as a disabling syndrome, which is relatively frequent across the general population, affecting 8% of adults in the United States, with an incidence of about 18 million people per year^1^. The International Association for the Study of Pain (IASP) defines chronic pain as pain experienced every day for 3 months over a period of 6 months or longer^2^. Chronic pain can be related to variety of medical conditions and commonly leads to a complex sensory and emotional experience with variety of features. Perception of chronic pain depends on the context and meaning of the pain, the physical, psychological, and psychosocial state of the patient^3^. The management of patients with chronic pain is considered to be one of the most difficult challenges in medicine^4^. Various chronic pain syndromes, such as post-stroke pain, trigeminal neuralgia, or phantom limb pain are highly resistant to pharmacological treatment^4^. Neuromodulation techniques have been increasingly used either as a substitute for surgical treatment or in addition to pharmacological therapy. Several conditions, such as essential tremor, Parkinson’s disease, dystonia, and psychiatric disorders have been successfully controlled with neuromodulation therapy^5^. Stimulation of brain structures for the treatment of chronic pain, however, have led to variable outcomes. Several trials reported successful neuromodulation of ventral posterior lateral nucleus (VC, Ventralis Caudalis) of thalamus, periventricular grey, and periaqueductal grey deep brain stimulation (DBS) with mean relief more than 50%^6,7^. Motor Cortex Stimulation (MCS) was introduced by Tsubokawa in 1991 as a treatment approach for patients with intractable pain, in response to growing frustration with inadequate DBS efficacy in this patient population^8^. Initial studies revealed that in contrast to stimulation of the of primary sensory cortex, which increases pain, stimulation of the motor cortex causes suppression of neuropathic pain^8,9^. Later MCS was found to be effective for various types of neuropathic pain (NP), such as trigeminal neuralgia, peripheral neuropathy, neuropathic pain after spinal cord injury (SCI), and others^10,11^. Over the last decade MCS has emerge as a promising alternative to pharmacological therapy and for patients with drug-resistant chronic pain syndromes^5,12^. Interestingly, until now, no standardized approach for MCS has been formed and there is no consensus regarding the surgical technique, electrode array implantation site, and optimal stimulation parameters^13^. The surgical technique, as well as target optimization varies between different centers with most of the institutions implanting epidural or subdural electrodes based on cortical anatomy or results of functional imaging. In order to identify cortical areas, which give the best clinical outcome with MCS and optimal initial stimulation settings, a trial stimulation was introduced at Mayo Clinic and was successfully used over last decade^14,15^. In this study we retrospectively evaluated the effect of ESCC in patients treated with the inclusion of a period of trial stimulation prior to permanent implant. The electrode locations with correction for brain shift were correlated with clinical outcome. In all cases the temporary electrodes were placed in the subdural space for initial trial stimulation and, then, were replaced with permanent electrode arrays for subsequent long-term stimulation. All procedures were performed off-label based on previously reported efficacy^14^. The outcome of motor and premotor cortex stimulation was analyzed and correlated with the position of the ‘most effective’ temporary electrode contacts and contacts on the permanent electrodes based on reconstruction of anatomical location.

## Results

### Medical records of nine subjects were acquired from Mayo Clinic database (***Table ******1***) from patients who underwent cortical electrode implantation procedure to treat chronic pain (***Fig. ******1***)

**Table 1:** Summary of initial clinical findings for patients in this study.

Subject number	Age	Sex	Main Diagnosis	Coexisting diagnosis	Pain duration (years)	Medications
1	66	m	Central post-stroke syndrome affecting left arm and leg	Stroke, hyperlipidemia, history of depression and anxiety, obesity, orthostatic hypotension, impaired fasting glucose	15	Clonazepam, dronabinol, gabapentin, venlafaxine
2	45	m	Phantom limb pain (left upper extremity)		5	Amitriptyline, gabapentin, DREZ surgery
3	83	m	Phantom pain (left upper extremity)		58	Gabapentin, duloxetine, amitriptyline
4	61	m	Left facial pain with a anesthesia dolorosa and SUNCT	DM type 2, hypertension	21	Indomethacin, venlafaxine
5	61	f	Right upper facial pain		3.5	Hydrocodone, baclofen, gabapentin
6	40	f	Right trigeminal neuralgia V2,3	History of non-psychotic major depression and anxiety, PTSD	1.8	Acetaminophen, lorazepam, oxcarbazepine, promethazine
7	55	f	Left trigeminal neuralgia and subsequent anesthesia dolorosa	Anxiety secondary	3.3	Anastrozole, baclofen, diazepam
8	61	f	Right trigeminal neuralgia		13.5	Hydrocodone, cyclobenzaprine, lamotrigine, topiramate
9	59	f	Chronic left greater right lower face and left parietal scalp pain	Laminectomy and L4-5 fusion	2.5	Oxycodone, gabapentin, lidocaine, diclofenac, methadone

*DM* diabetes mellitus, *PTSD* posttraumatic stress disorder, *DREZ* dorsal root entry zone.

**Figure 1:**
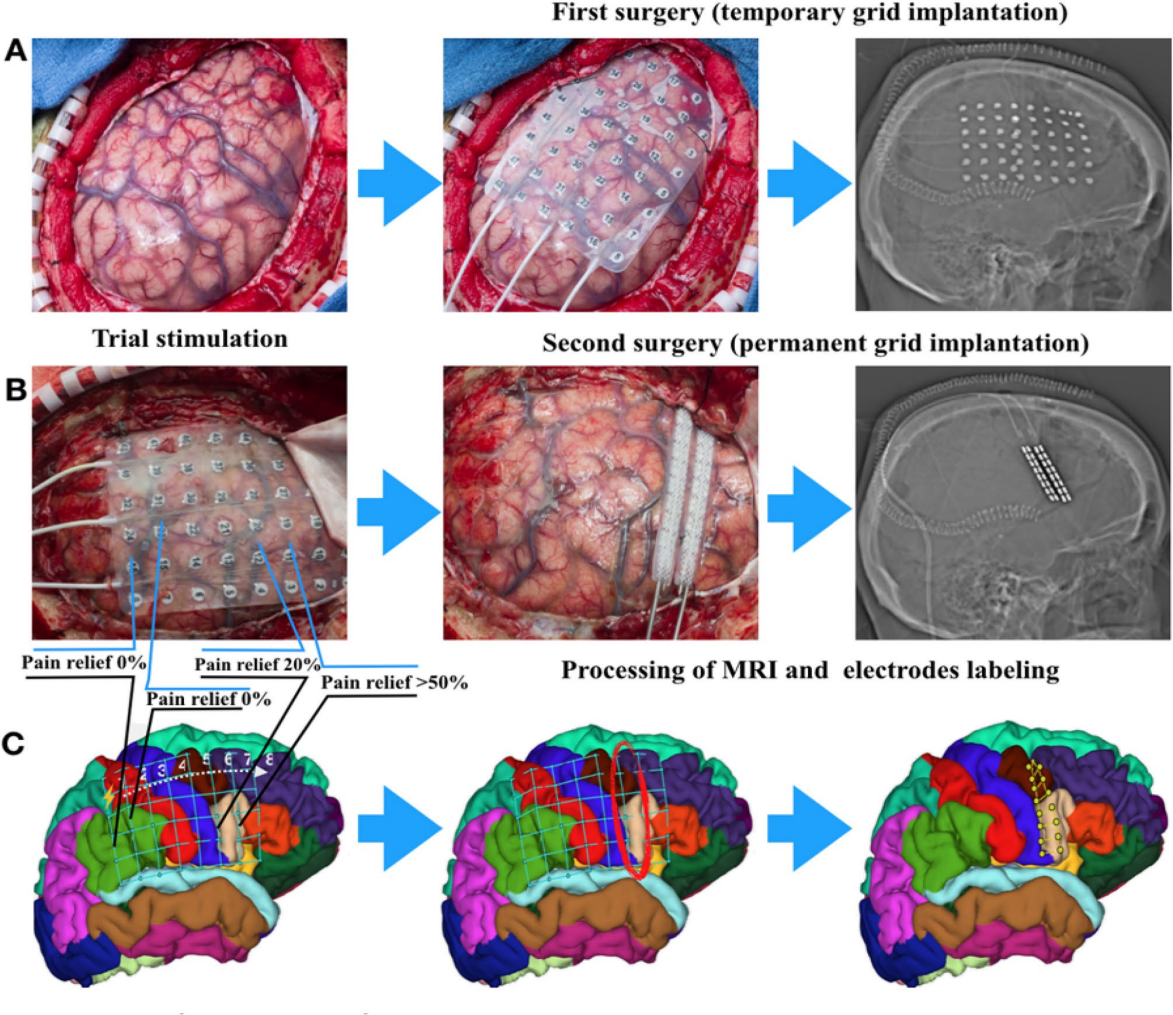
Surgical intervention and mapping. (**A**) Surgery for temporary grid placement in order: craniotomy; 6 × 8 temporary grid placement; X-ray/CT-scan representative for temporary grid position. (**B**) Trial stimulation for target optimization, with following permanent grid implantation over identified area. X-ray/CT-scan representative for permanent grid position. (**C**) Further image processing to reveal electrodes position over exact cortex area according to Desican-Killiany atlas.

**Figure 2:**
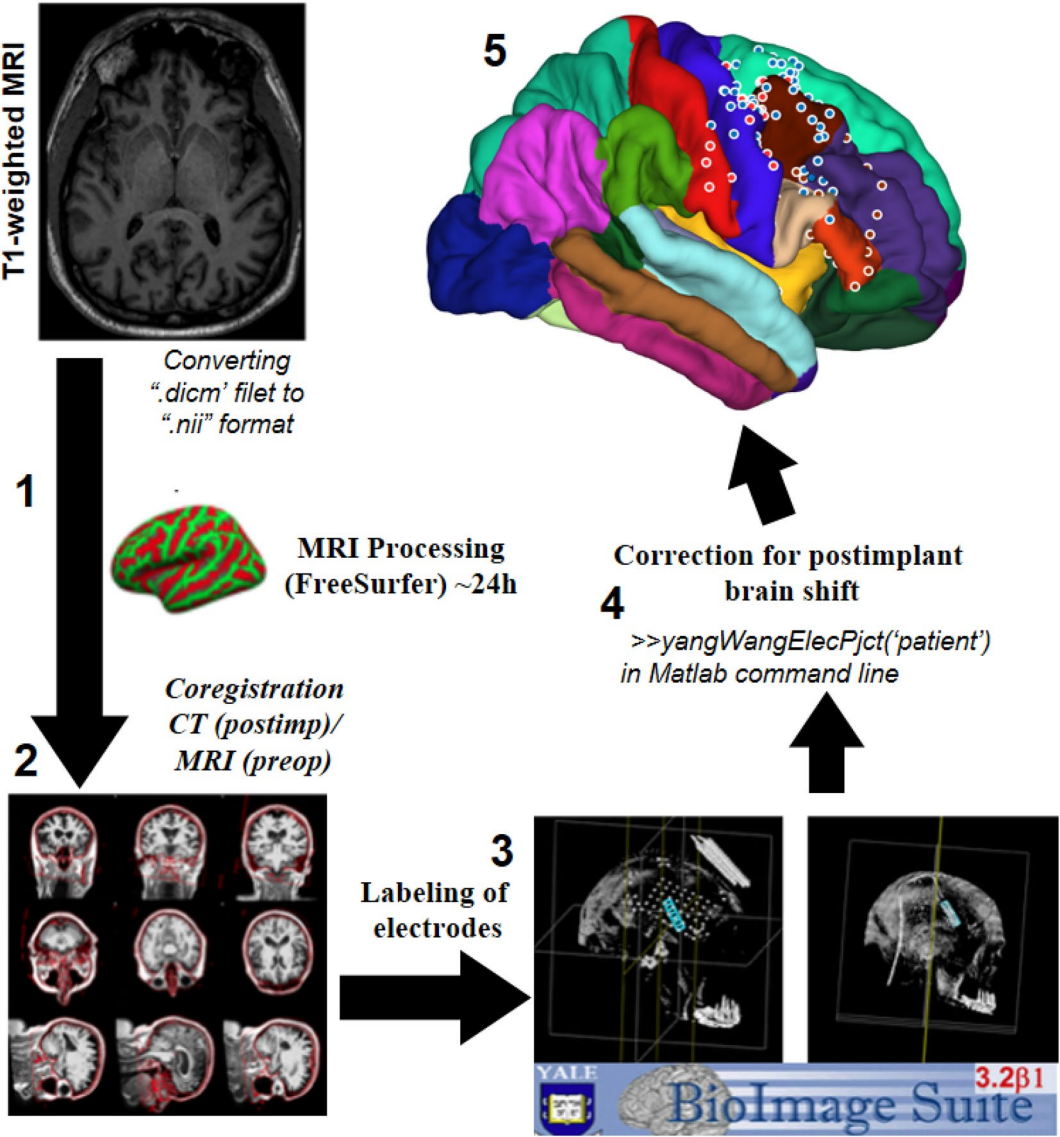
Algorithm of the image processing. (1) Processing of the T1-weighted MRI image using freesurfer(extraction of the pial surface and leptomeningeal surface, segmentation of brain structures); (2) Coregistration of the postimplant CT scan with processed MRI using iELVis coregistration tool; (3) Labeling of electrodes on coregistrated CT using Bioimage Suite 3. (4) Postimplantation brain shift correction using iELVis Yang, Wang et.al. tool. (5) Creating images of electrode locations. ( Source: http://ielvis.pbworks.com/).

Algorithm of the image processing and post-implantation brain shift correction described in methods and outlined on Fig. 2.

### Trial stimulation

Retrospective review of programming records demonstrated that after surgical implantation of the permanent grid, all subjects in this study (n = 9) reported a "honeymoon effect" with significant improvement in pain during the first one to two days after surgery without active stimulation. Because of ‘honeymoon effect’, trial stimulation was delayed until the pain level exceeded 5/10 on the Numeric Rating Scale (NRS). Parameters of stimulation (frequency, pulse width, and stimulation intensity) were adjusted individually during trail stimulation and permanent stimulation and varied depending on pain reduction and side-effects. Initial settings were: 40 Hz, 450 µsec, 0–4 V. In two cases, stimulation frequency was increased up to 100 Hz (subjects 4 and 9), when the initial settings did not improve pain. Trial stimulation was started on day 1 (n = 3), 2 (n = 1), 3 (n = 3), or day 4 (n = 1) after temporary grid placement. In all subjects except one, the initial trial stimulation reduced the pain intensity in more than 50%, although one subject (subject 9) didn’t show reduction in pain and after trial stimulation his pain level was 8/10, comparing with 9–10/10 at baseline before implantation. The mean pain level by the end of the trial stimulation was 2.16 ± 2.39 (n = 9). Based on results of trial stimulation the areas of stimulation with the most effective pain control were identified as a target for permanent grid placement (Fig. 1). Trial stimulation was also helpful in evaluation of potential side effects of ESCC, i.e. problems with speech, muscles spasm or twitches, focal seizures, and in some cases increased pain (Table 2). For all cases, the final target for implantation of permanent grid was adjusted based on results of the trial stimulation.

**Table 2:** Results of trial stimulation with temporary and permanent grid.

Subject number	Pain type	PO-pain (NRS)	Trial stimulation				Permanent stimulation		Side effects T-during trial stimulation P-during permanent stim	Permanent electrode placement (cortex)
			Start		End					
			Day	NRS	Day	NRS	7–14 days (NRS)	6–12 m follow-up*		
1	CPSleft	9–10	3	5	8	2	2	2	Focal seizure, intermittent dysarthria (T)	Motor Premotor
2	PhPleft	8–10	1	4–5	5	2	2	2–3	Epidural, subdural hematoma (T)	Motor Premotor
3	PhPleft	5–10	1	5	7	0–1	0–1	1	Headache	Motor Premotor
4	RFP	8–9	3	8	8	3	3	5	Intermittent aphasia, incisional pain (T\P)	Motor Premotor
5	LFP	8–9	1	5	3	3–4	3–4	4–5	Intermittent facial twitching (T)	Motor Premotor
6	RFP	9–10	4	6–7	6	0	0	5	Intermittent right side seizures, aphasia(T)	Motor Premotor
7	LFP	6–10	2	7	7	0	0	0	Throat spasm, whole body shaking w/o loss of conscious (T)	Premotor
8	RFP	9–10	3	7	8	0–1	0–1	0–1	Intermittent aphasia(T)	Premotor
9	LFP	9–10	0	5	3	8	8	8–10	Subdural hematoma (T)	Sensory Motor Premotor

PO pain-preoperative pain level; Trial stimulation—mapping with temporary grid stimulation: start day—days after temporary grid implantation; start NRS—pain by the trial stimulation beginning; end day—trial stimulation end after temporary grid implantation; end NRS—pain by the end of trial stimulation; Chronic stimulation—stimulation with permanent grid: *NRS score within 6–12 months of observation is considered as an average pain level during this period that achieved after stimulation parameters adjustment and adequate battery charge. CPS left—central poststroke pain syndrome with left-side clinical presentation; PhP left–left side phantom pain, RFP–right fascial pain; LFP–left fascial pain.

### Chronic stimulation

**Figure 3:**
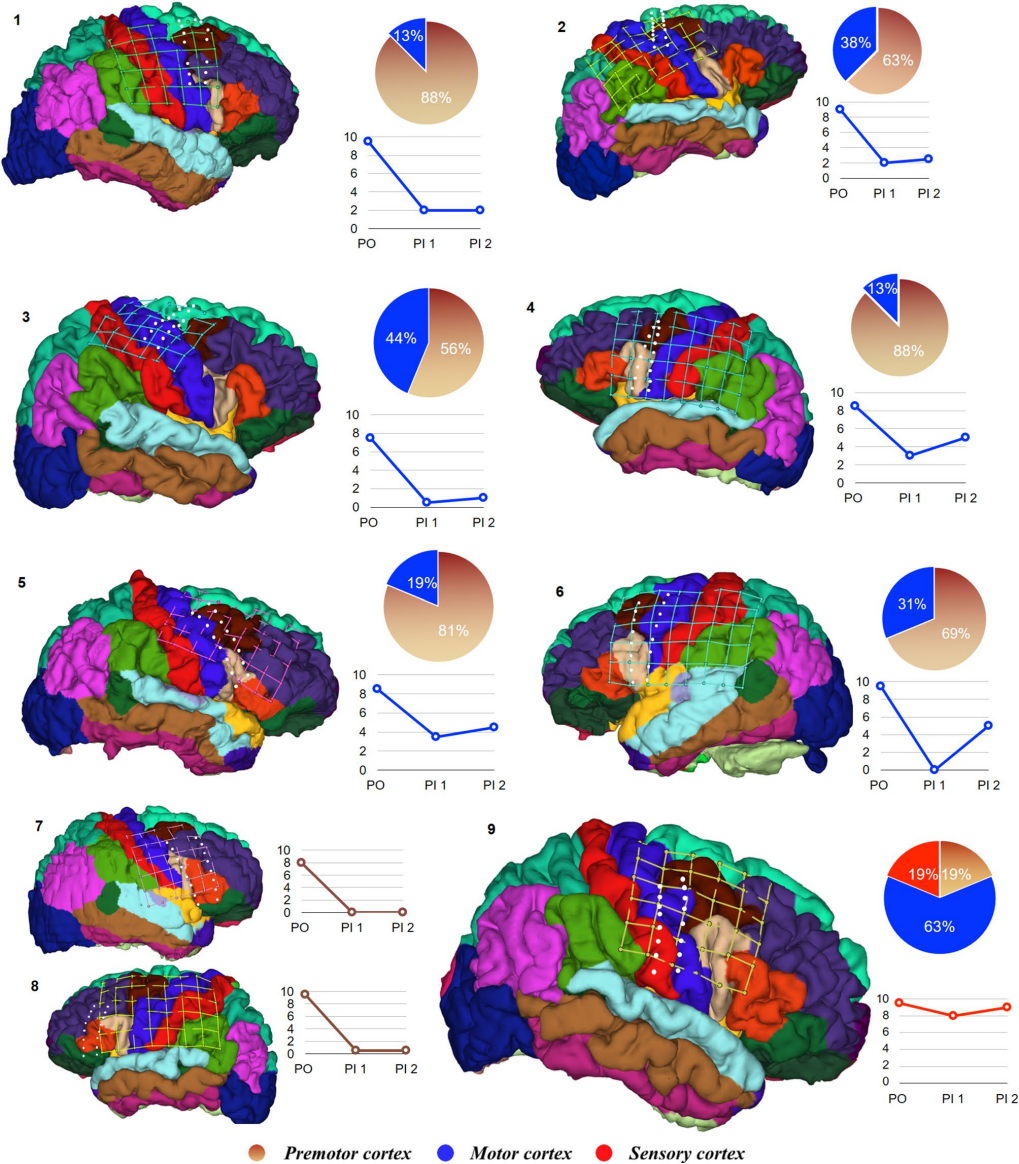
Pain reduction effect of chronic subdural cortex stimulation. Reduction of pain with chronic subdural cortical stimulation presented here in three groups, based on electrodes location. 1st group: patients 1–6 with permanent grid implantation over both premotor and motor cortex; 2nd group: patients 7–8 with permanent grid implanted over premotor cortex; 3rd group: patient 9 for whom permanent grid implanted over motor, premotor and sensory cortex. Pie charts illustrate proportion of electrodes over particular cortex area. Line charts represent variation of pain level in patients after permanent grid implantation. PO—preoperation pain level; PI—permanent grid post-implantation pain level (PI1- within 2 weeks after permanent grid implantation; PI2—pain level during 6 months to 1-year period of observation).

**Figure 4:**
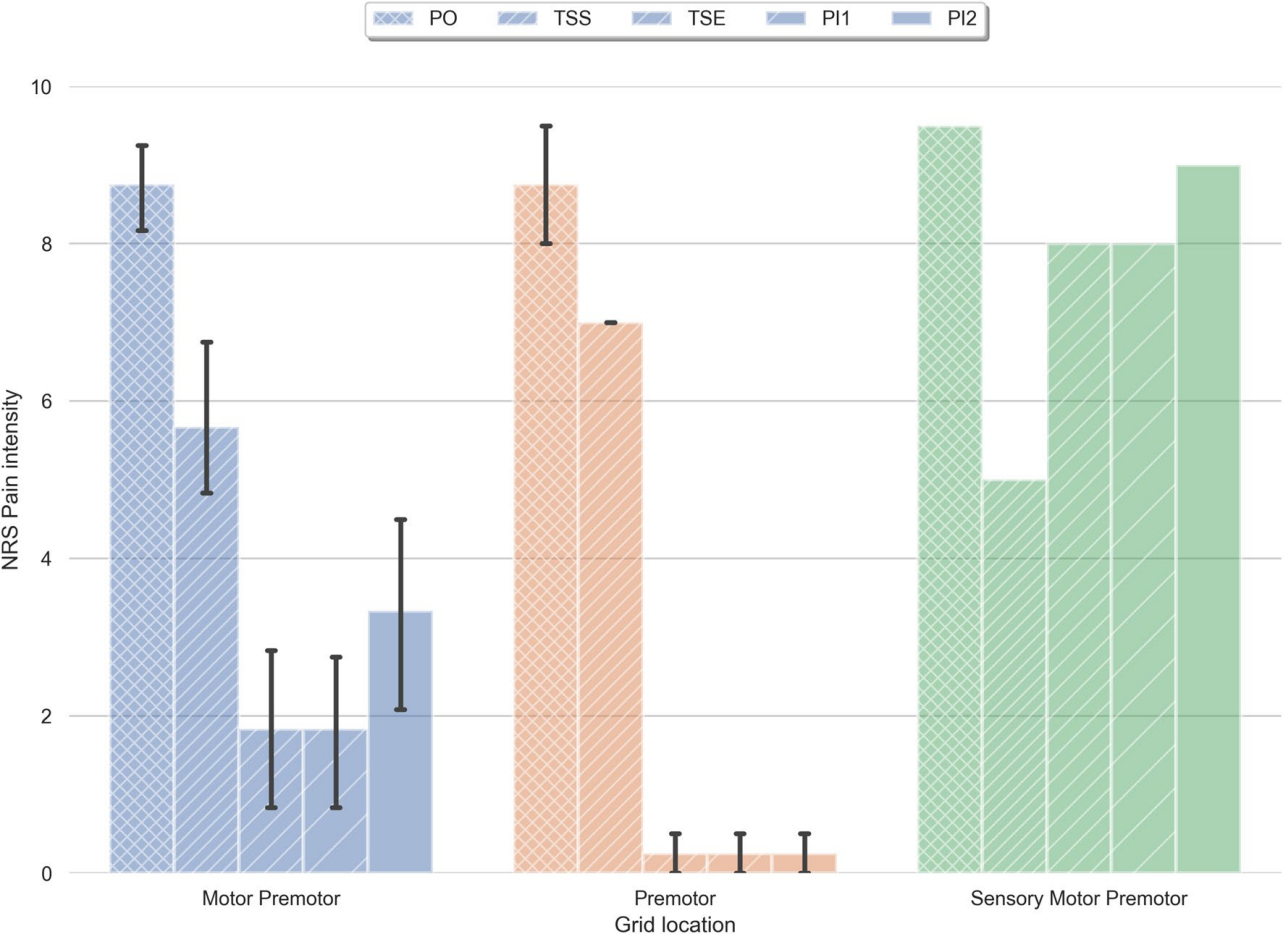
Comparison of ESCC effects in three groups based on electrodes location. Blue—electrodes implanted over motor and premotor cortex (n = 6); Brown—electrodes implanted over premotor and prefrontal cortex (n = 2); Green—electrodes implanted over sensory, motor, and premotor cortex (n = 1). PO—preoperative pain level (NRS); TSS (trial stimulation start)—pain level when trial stimulation was started; TSE (trial stimulation end)—pain level by the end of trial stimulation; PI1 (post-implantation 1)—permanent grid post-implantation pain level within 2 weeks; PI2 (post-implantation 2)—permanent grid post-implantation pain level that had been achieved after adjustment of stimulation settings within 6–12 months of observation. Means ± standard deviation of the mean.

**Figure 5:**
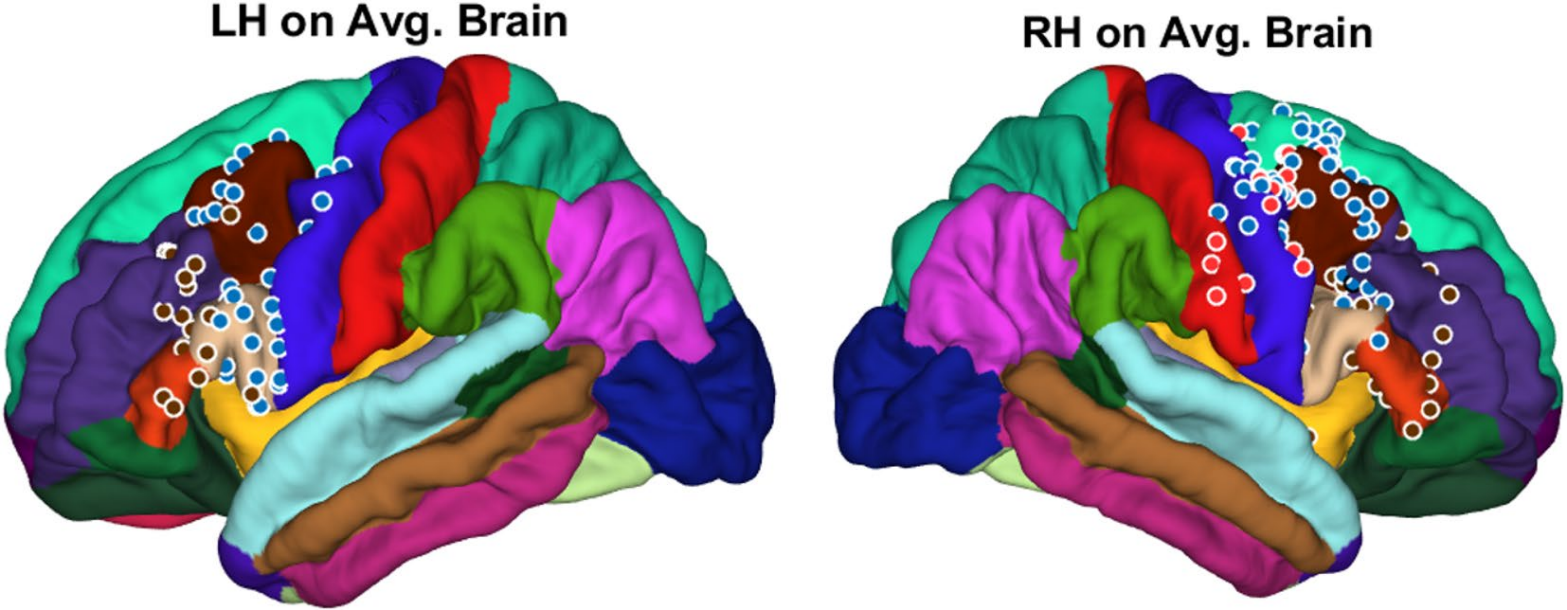
Location of permanent grid electrodes on average brain model. 1st group (blue dots): subjects 1–6 with permanent grid implantation over both premotor and motor cortex; 2nd group (brown dots): subjects 7–8 with permanent grid implanted over premotor cortex; 3rd group (red dots): subject 9 whom permanent grid implanted over sensory, motor, and premotor cortex.

All subjects then underwent implantation of the permanent electrodes (2 × 8 spinal leads) centered over the most effective leads of the initial trial stimulation, except subject 9, who developed postoperative hematoma. After recovery from the 2nd surgery, they were evaluated and discharged with follow-up visits and evaluations based on regular reports. Based on results of retrospective analysis, patients with electrodes implanted over motor or motor and premotor areas (n = 6), yielded at least 70% pain reduction on the average and stated stable pain relief up to 0–4/10 on the NRS (Fig. 3). Patients with permanent electrodes placed over the premotor area (n = 2) yielded significant pain reduction and stated pain relief up to 0–1 on pain scale that was better compare to other patients (Figs. 3, 4). We identified one patient (Subject 9) with permanent electrodes covering the motor, pre-motor cortex, and also sensory cortex, who did not demonstrate significant pain relief (~ 5%). This patient had a postsurgical hematoma with the following cerebral edema, and, because of this surgical complication, permanent grid was implanted after hematoma evacuation without completion of the trial stimulation. Shifting of the electrode as a result of reduction in cerebral edema lead to abnormal electrode position. Six months to one-year follow-up after permanent electrodes implantation in this subject demonstrated that stimulation with permanent electrodes, pain increased up to the preoperative level (Fig. 4). In six subjects with electrodes located over premotor and motor area, pain reduction slightly decreased by 15%. However, two subjects with electrodes placed on premotor area did not show significant fluctuation in pain level over time (Table 2). At six-month follow up visit, all subjects who had a significant pain relief during trial stimulation demonstrated stable pain control (Fig. 4). In contrast, subjects, who had improvement less than 50% from the pre-operative level, demonstrated some level of decline in effect of ESCC to control chronic pain (Table 2). The analysis of the effect of ESCC to alleviate chronic pain in correlation with the location of permanent electrodes suggests that the neuromodulation of premotor cortical area may be as beneficial as, or better than primary motor cortex stimulation. The reconstruction of permanent electrodes location on average brain model demonstrated that subjects with significant pain relief have electrodes primarily on pre-motor cortex (Fig. 5).

## Discussion

The surgical treatment of neuropathic pain with ESCC has been evolving over last decades, starting from the first implantation, reported by Tsubokawa and colleagues^8^. Most of the centres currently using image guidance with MRI to localize the region of the pain, i.e. in case of facial pain—anterior to the central sulcus at the level of the inferior frontal sulcus^8^. This information then loaded into a neuronavigation workstation to direct the most appropriate location for the incision and craniotomy. Typically, the specific region of motor cortex is identified through intraoperative somato-sensory evoked potentials (SSEP) and confirmed by trial intraoperative stimulation^8,10^. In addition, TMS can be useful for non-invasive trial stimulation and might potentially be considered as an assessment tool for cortical stimulation efficiency^16^. The surgical technique, however, varies significantly between institutions, with most centers placing electrodes in the epidural space. Epidural placement has several advantages including lower operative risk and shorter operative time, however, epidural electrode placement often leads to a scarring around the electrodes, resulting in higher electrode impedance and consequent declines in stimulation efficacy over time^17,18^. Epidural stimulation can also lead to less localized stimulation and may require a higher stimulus intensity, which usually results in shorter battery life. Epidural stimulation may also induce pain by direct activation of the dural pain fibers, limiting therapeutic effect of stimulation^4,14,19^. In a computer simulation study, Kim et al. compared the efficacy of both epidural and subdural placement and reported that the effective volume and depth of pulse penetration is significantly higher with subdural stimulation compared to epidural^20,21^. Placing stimulating electrodes in the subdural space routinely over last ten years we have shown consistent results with minimal surgical complication^14,15^.

Regardless of the long history of ESCC, the mechanisms responsible for alleviating of chronic pain are largely unknown. In animal studies it has been demonstrated that after deafferentation of reticular thalamic nucleus and ventral posterior lateral thalamus, normal thalamocortical circuitry activity shifts from high-threshold tonic firing to low-threshold theta-range oscillatory bursts^22^. This transformation further leads to a decrease in the excitatory input to the reticular thalamus and their subsequent hyperpolarization and low-frequency bursting that induces rhythmic discoordination of the thalamocortical loops in theta frequency band^23^. Based on these observations it was proposed that cortical stimulation may inhibit the hyperactivity of the thalamus and particularly the sensory nuclei of the thalamus that exhibit chronic pain-induced hyperactivity with increased spike density^8^. In fact, outcomes of multiple trials suggest that the mechanisms of neuropathic pain that respond to the cortical stimulation may have a final common pathway of deafferentation at the different levels of the sensory system. Later, it was shown that cortical stimulation may initially activate the axons that run horizontally in the precentral gyrus, parallel to the surface of the cortex. These cortico-cortical projections directed from primary motor to primary sensory cortex, and travel in Layer I, making them easily accessible for stimulation^14^. These results altogether support the commonly accepted hypothesis that during neuropathic pain, most of the cortical inputs tend to balance pathological thalamocortical oscillations and may also facilitate following reorganization in these areas, while cortical stimulation targeting the same mechanisms could further compensate pathological oscillations and related neuropathic pain. The brain areas with relatively higher degree of functional connectivity and plasticity, like premotor and motor cortical areas, could be particularly effective in compensation of central deafferentation. Multiple studies suggest that cortical stimulation can lead to reinforcement of intracortical GABAergic inhibition, increased secretion of endogenous opioids in various structures and more specifically, the cingulate cortex and periaqueductal gray matter (PAG)^24,25^. It was also found that the density of opioid receptors binding in the brain is correlated with postoperative pain relief with cortical stimulation in patients with chronic pain^25^. Another mechanism of ESCC could be related with activation of cortical and mesencephalic areas involved in the emotional appraisal of pain, particularly insula, cingulate, and orbitofrontal cortex^26,27^.

Only few studies have explored the effect of cortical stimulation on various pain syndromes (Supplementary Table 1) with total of over few hundreds participants evaluated^4^,^8^,^10^,^24^,^28^,^30^–^32^,^34^,^52^–^54^. Most of reports with MCS are focusing on trigeminal neuralgia and post stroke pain treatment. Trigeminal neuralgia generally showed a good response to cortical stimulation with more than a half of the patients receiving significant pain relief^10,22,28^. Several recent reviews indicate that 75–85% of patients have at least 50% reduction of trigeminal pain with motor cortex stimulation^9,21^. Results however, vary between the centers^4,29^. In contrast with trigeminal neuralgia, there is a lack of clinical trials on cortical stimulation for phantom limb pain with only few case-reports and mixed trials with small number of patients. Recently we reported an improvement of phantom limb syndrome with cortical stimulation in two subjects along with other studies demonstrating effect of cortical stimulation on phantom limb pain^15,30,31^. Currently, the implantable neuromodulation systems for MCS in patients with chronic pain are considered “off label” which significantly limits further exploration of this technique^32^. MCS can lead to several complications, typical for most craniotomies, such as infection and hemorrhage (Supplementary Table 1). Previously reported findings indicate on potential side effects directly related to the cortical stimulation, including intermittent dysarthria, aphasia, throat spasm, focal seizure, and intermittent facial twitching. In all cases these side effects were terminated with reducing intensity or termination of the stimulation. The seizure threshold does appear to respond to standard anticonvulsants (e.g. levetiracetam or fosphenytoin) that were used in this study^26,30,33,34^. Henderson et al. reported that seizures were associated primarily with stimulus rates between 70 and 90 Hz and patients who experienced seizures did not develop chronic epilepsy^35^. Other reported surgical complications include hematoma, as a consequence of subdural implantation, and a headache that could be related to craniotomy and liquorrhea.

**Figure 6:**
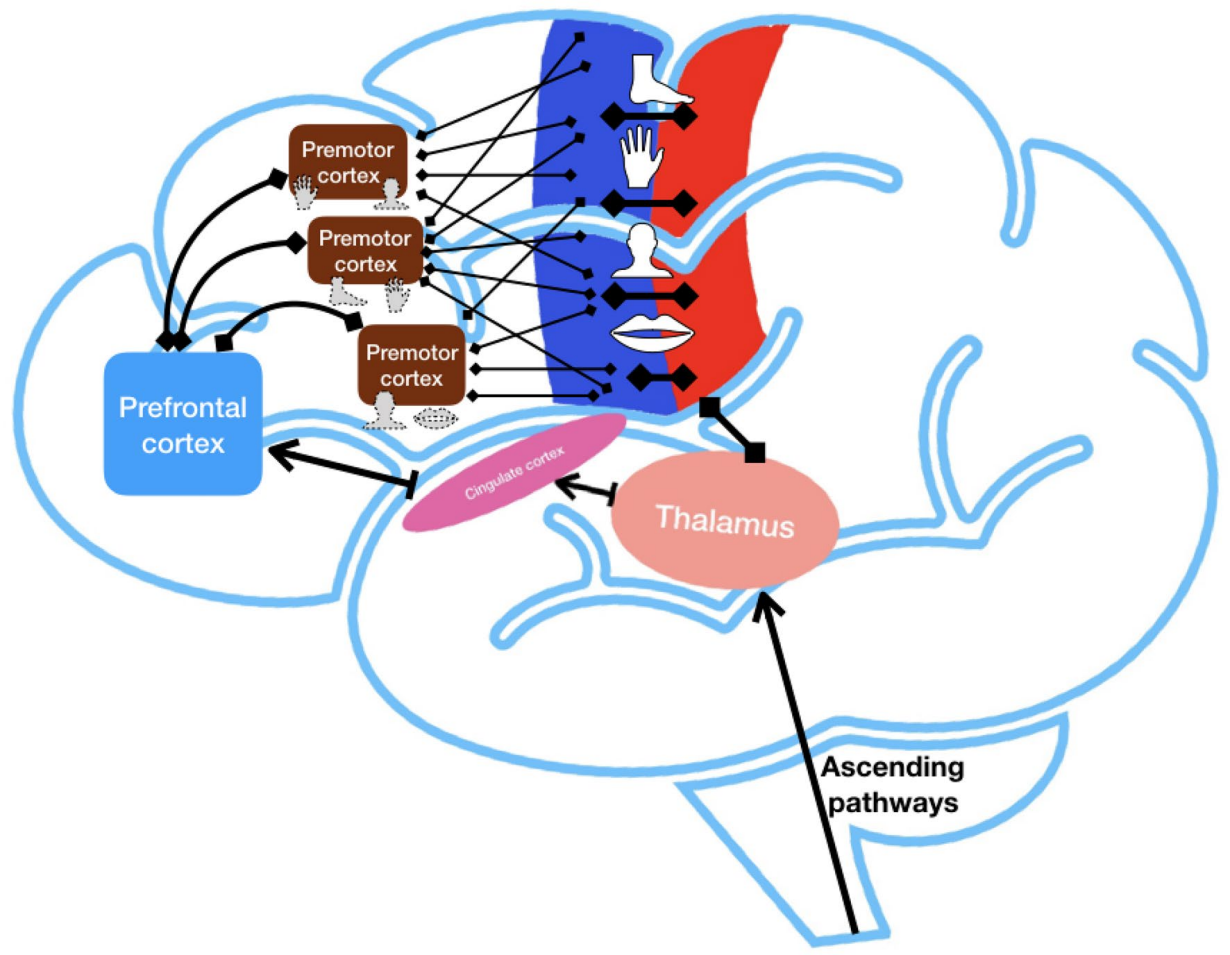
Hypothetical organization of network responsible for sensorimotor integration and pain processing (prepared using Keynote, Apple).

Summarizing these results, we can outline two key outcomes of this study. The first outcome indicates on *specific role of electrode location in the efficacy of cortical stimulation to control chronic neuropathic pain, and particularly the effect of pre-motor cortex stimulation.* Most of the previous studies were focused on neuromodulation of the motor cortex, after initially negative experience with S1 stimulation^8,10,20^. The effect of premotor cortex stimulation was not studied and remained largely unknown. In contrast to a somatotopic organization (motor homunculus), premotor cortex is likely organized in a functional manner, where overlapped regions represent different motor patterns^36–38^. This organization has been supported by several studies in animals, and indicates that the areas of cerebral cortex controlling specific part of the body in pre-motor cortex may have more diffuse and overlapped representations compared to M1, i.e. related to complex sensorimotor functions with involvement of multiple body parts and may have higher cortical plasticity^35,39^. This specific organization was primarily suggested by animal studies and still needs to be confirmed in humans. Recent works suggest that premotor cortex along with primary motor cortex, primary sensory cortex, and prefrontal cortex are organized in a neuronal network responsible for complex control of movements and sensorimotor integration (Fig. 6)^35,39–41^. Specific mechanisms of this organization and functioning of this network still need to be investigated and may further facilitate development of new strategies for treatment of neuropathic pain.

The second outcome of this study indicates on *importance of trial stimulation for target optimization.* According to these results, initial reduction in pain, observed during trial stimulation, was consistent during chronic stimulation for most of the tested subjects. Those who did not respond to trial stimulation, in fact, did not improve, even after multiple adjustments. These findings suggest that trial stimulation provides important information for positioning of permanent electrodes and helps to assess individual response to initial stimulation settings. Trial stimulation started after the ‘honeymoon period’, when the pain is returning to the baseline, can help to avoid misinterpretation of results of trial stimulation. The main disadvantage of trial stimulation with temporary grids is surgical side effects, i.e. increased risk of hematoma and loss of CSF. In combination, this leads to the shifting from initially planned position and may result in displacement of the permanent grid that we observed in subject 9. Retrospective analysis of DBS implantations has been performed previously for evaluation of the electrode shift and did not find significant displacement with postoperative intracranial air volume up to 35 cm^3^, although, DBS electrodes are less affected by the brain shift due to their deeper locations^42^.

In conclusion, the results of this study demonstrate the first case-series report with subdural stimulation of the mixed motor and pre-motor cortical areas (n = 6) and solely pre-motor cortex (n = 2). Results demonstrate improvement in chronic neuropathic pain with pre-motor cortex stimulation from 8–9 to 0, compare to more moderated improvement with mixed motor and pre-motor cortex stimulation from 8–9 to 1–4 on NRS scale. The results of this study suggest that modulation of neural networks with subdural ESCC is beneficial and provides control of neuropathic pain when located on motor and particularly on pre-motor areas. Due to low number of subjects in the second group, these results will require future confirmation. The most important and novel finding of this study is that pre-motor cortex stimulation could be is at least as beneficial as motor cortex stimulation and can be considered for future therapy with ESCC. In cases of complex pain syndromes, i.e. thalamic pain, regional pain syndrome, or spinal cord injury-associated pain, pre-motor cortex stimulation could provide additional benefits through the coverage the wider functional area. Another important finding of this study is that trial stimulation for target optimization can significantly improve the outcome of ESCC. Future differentiation of specific mechanisms related to effect of ESCC could improve pain control in cases when access to the motor cortex is limited or the effect of stimulation is insufficient.

## Methods

### Patient selection

For trial stimulation subjects initially underwent implantation of a temporary grid of electrodes (6 × 6 or 8 × 6, based on expected size of testing area of the cortex) (Fig. 1A). After completing the craniotomy, the central sulcus was identified as well as the cortical region representing the primary sensory cortex. Preoperative functional MRI (fMRI) for tongue tapping and face brushing was implemented to aid in localization of these regions. Then, a temporary grid was placed in the subdural space. Data were merged with preoperative imaging in the neuronavigation workstation to guide the surgical procedure. Following placement of the chronic permanent electrode, the leads were tunneled, externalized, and connected to an external stimulus generator for trial stimulation monitoring in the intensive care unit (Fig. 1B). All patients underwent postoperative CT scan to localize the position of the grid. The initial assessment with trial stimulation using a temporary grid of subdural electrodes to optimize targeting and validate the effect of stimulation before placing the permanent electrodes was started after the initial ‘honeymoon period’ had worn off (generally in about 24 h), and when pain had increased at least to 6/10 on the NRS. The mean duration of the trial stimulation was usually 4–7 days, while patient was in the intensive care unit under continuous monitoring. Factors affecting the duration of trial stimulation included the duration of the ‘honeymoon period’, number of contacts on the temporary grid, latency to response with stimulation changes, stimulation-induced seizures, and clinical scheduling. Patients and/or nursing staff maintained an hourly pain journal to provide a guide for response to changes in stimulation parameters. Initial parameters for temporary grid trial stimulation at the motor and pre-motor cortex were: pulse width of 450 μs, rate of 40 Hz, intensity 0–4.0 V, selected as initial settings based on previous findings^14,15^. Subjects were maintained on seizure prophylaxis with fosphenytoin throughout trial stimulation (1000 mg once during surgery and 300 mg daily during trial stimulation) and following the permanent implantation (also at 300 mg/d) for one week. Each column of the implanted subdural electrode array was tested with cathodal stimulation. If successful with the patient reporting a 50% reduction in pain (or reduction below 5/10 with baseline 8–10/10), the patient returned to the operating room for implantation of permanent electrode array for chronic stimulation (2 × 8 spinal leads, Medtronic Leads 977C265, Medtronic USA Inc) (Fig. 1) connected to an internal pulse generator positioned in the (usually ipsilateral) subclavicular region. The final target for implantation of the permanent grid was adjusted based on the results of the trial stimulation. All subjects were evaluated every few weeks during the following up visits while chronically stimulated. All procedures were approved by the the Mayo Clinic Institutional Review Board (IRB).

### Image processing for reconstruction of the electrode contact locations

As a part of each patient's routine presurgical evaluation, a preoperative T1-weighted volumetric MRI without contrast was acquired, typically a sagittal magnetization-prepared rapid gradient echo (MPRAGE) (voxel dimensions 0.86 × 0.86 × 0.9 mm, 3 T field strength), although additional coronal and axial MPRAGE and spoiled gradient recalled echo images with similar resolution were conducted as well. The preoperative MRI was converted using a DICOM to NIfTI conversion tool for Matlab and preprocessed in FreeSurfer to segment brain structures, extract the pial surface, extract the leptomeningeal surface (i.e., a smoothed pial surface), and map the patient's cortical surface to the FreeSurfer average cortical surface (Fig. 2)^43,44^. As part of this process, each patient's cortical surface was mapped to the Desikan-Killiany brain atlas, which assigns each neocortical vertex to 1 of 35 areas based on gyral morphology^44^. After grid implantation, high-resolution CT images were acquired to confirm electrode placement. The post-implant CT volume was co-registered to the preoperative MRI using the FMRIB's linear image registration tool algorithm (ct2mri.sh command line tool) included in FSL via a 6 degrees of freedom affine transformation that maximized the mutual information between the 2 volumes^45,46^. Co-registered image volumes were visually inspected for accuracy. After co-registration, electrodes were manually labeled at the coregistered CT volume imported into BioImage Suite 3.0^47^. The cortical surface and electrode positions in the postimplant images were analyzed. Functional mapping (motor and sensory cortex) was not performed in this study due the absence of data on the importance of this approach in adjustment of the permanent cortical grid location. As presented earlier, the initial electrode position on CT images may shift due to loss of CSF during surgery, cerebral edema or surgical complications, such as hematoma^48,49^. To compensate for potential brain shift, subdural grid and strip electrode coordinates were projected to the leptomeningeal surface using an inverse gnomonic projection method described by Yang et al. (“yangWangElecPjct (‘subject’)” in Matlab command line)^50^. The surface under each grid was approximated as part of a larger sphere, and the algorithm iteratively adjusted the projection of the grid plane onto the sphere to minimize the difference between the projected and known electrode geometry. Subdural strip electrodes were assigned to the nearest leptomeningeal surface vertex. The brain shifting correction was done in MATLAB (Mathworks Inc., Natick, MA) via iELVis package^51^. iElVis was also used to identify the anatomical location of the permanent electrodes. The result of this processing is the grid location labeled at the hemisphere cortical surface mapped to the Desikan-Killiany brain atlas with defined cortex areas (“plotMgridOnPial (‘patient’)” in Matlab command line) (Fig. 2)^44,50^. All results are reported as means ± standard deviation of the mean.

### Informed consent

Informed consent, including the discussion of off-label use of FDA approved devices, was obtained from all individual participants included in the study. Following our practice of subdural cortical stimulation performed off-label, the patients underwent a two-step procedure that involved implantation of a trial electrode array followed by permanent spinal leads and an internalized pulse generator.

### Ethical statement

All procedures performed in studies involving human participants were in accordance with the ethical standards of the institutional and/or national research committee and with the 1964 Helsinki Declaration and its later amendments or comparable ethical standards. This project was limited to a retrospective review of information from medical records within a group of patients who accepted MN research authorization. All data were analyzed anonymously with the approval of the Institutional Review Board (IRB) of our institution (ID# 18-006449) and in accordance to the usual policies and safeguards enforced by the Department of Health Science Research to protect the confidentiality of the patient record.

### Supplementary Information

Supplementary Information.

## Data Availability

Data collected for the study were deidentified and will be available with publication.
