# Author Correction: Pre-motor versus motor cerebral cortex neuromodulation for chronic neuropathic pain

**DOI:** 10.1038/s41598-024-65260-5

**Published:** 2024-07-02

**Authors:** Igor Lavrov, Timur Latypov, Elvira Mukhametova, Brian N. Lundstrom, Paola Sandroni, Kendall Lee, Bryan Klassen, Matt Stead

**Affiliations:** 1 Department of Neurology, Mayo Clinic, Rochester, MN USA; 2 Department of Biomedical Engineering, Mayo Clinic, Rochester, MN USA; 3 Institute of Fundamental Medicine and Biology, Kazan Federal University, Kazan, Russia; 4 Skolkovo Institute of Science and Technology, Moscow, Russia; 5 Division of Brain, Imaging, and Behaviour Systems Neuroscience, Krembil Research Institute, Toronto Western Hospital, University Health Network, Toronto, ON Canada; 6 Institute of Medical Science, Faculty of Medicine, University of Toronto, Toronto, ON Canada; 7 Department of Neurologic Surgery, Mayo Clinic, Rochester, MN USA

Correction to: *Scientific Reports* 10.1038/s41598-021-91872-2, published online 16 June 2021

The original version of this Article contained an omission in the name of Brian N. Lundstrom, which was incorrectly given as Brian Lundstrom.

The original Article has been corrected.

