# Supplementary material for: Pre-motor versus motor cerebral cortex neuromodulation for chronic neuropathic pain
Source: Sci Rep. 2021 Jun 16;11:12688. doi: 10.1038/s41598-021-91872-2 (PMC8209192; doi:10.1038/s41598-021-91872-2)
Supplement: Supplementary file 1 — Supplementary Information. [file 41598_2021_91872_MOESM1_ESM.docx]

**Pre-motor vs. motor cerebral cortex neuromodulation for chronic neuropathic pain**

**Igor Lavrov**^1,2,3*^, **Timur Latypov**^4,5^**, Elvira Mukhametova**^3^**, Brian Lundstrom**^1^**, Paola Sandroni**^1^**, Kendall Lee**^6^**, Bryan Klassen**^1^ **& Matt Stead**^1^

Author affiliation:

^1^Department of Neurology, Mayo Clinic, Rochester, MN, USA

^2^Department of Biomedical Engineering, Mayo Clinic, Rochester, MN, USA

^3^Institute of Fundamental Medicine and Biology, Kazan Federal University, Kazan, Russia

^4^Division of Brain, Imaging, and Behaviour Systems Neuroscience, Krembil Research Institute, Toronto Western Hospital, University Health Network, Toronto, Ontario, Canada

^5^Institute of Medical Science, Faculty of Medicine, University of Toronto, Toronto, Ontario, Canada

^6^Department of Neurologic Surgery, Mayo Clinic, Rochester, MN, USA

^*^Corresponding author: I. Lavrov (igor.lavrov@gmail.com )

| **№** | **Author, year** | **N of patients** | **Pain type** | **Success on TS**  **#** | **Long-term success/**  **Pain relief(control)** | **Side effects** |
| --- | --- | --- | --- | --- | --- | --- |
| 1 | Tsubokawa et al., 1991 ^S1^. | 7 | CPS | NA | ~71/100% | ·· |
| 2 | Meyerson BA et al., 1993 ^S2^. | 10 | TN; CPS; PPTN | 90% | 50% /~60-90% | Short-lasting generalized seizures during TS |
| 3 | Ebel H et al., 1996 ^S3^. | 7 | TN | 1 TS | 86% / >50% | Focal seizure |
| 4 | Nguyen JP et al., 2000 ^S4^ | 32 | TN; CPS; PPTN; SCI | NA | ~70% / 52% | Subcutaneous infection; incisional pain; epidural hematoma |
| 5 | Carroll D et al., 2000 ^S5^ | 10 | TN; BPI; PPTN; | 50% | 40% / > 50% | Local tenderness over the electrode site |
| 6 | Roux FE et al., 2001^S6^. | 1 | PhP | NA | Pain relief 70% | ·· |
| 7 | Rasche D et al., 2006 ^S7^ | 17 | TN; CPS | 47% | 47% / >50% | Intraoperative seizure, wound infection, speech arrest (3 months) |
| 8 | Delavallee M et al., 2008 ^S8^ | 8 | TN; CPS; PPTN; BPI | NA | 50%/>80%, mean pain relief  ~66% | Partial motor and generalized seizures, arachnoiditis |
| 9 | Velasco F et al., 2008 ^S9^. | 11 | CPS; BPI | 73% | Mean pain relief ~63% * | ·· |
| 10 | Lefaucheur JP et al., 2009 ^S10^. | 16 | TN; PhP; PPTN; BPI | ~60% | 60% / >50%, mean pain relief - 48% | ·· |
| 11 | Raslan AM et al., 2011 ^S11^ | 11 | TN | 73% | ~45% /> 50% | ·· |
| 12 | Pereira EAC et al., 2015 ^S12^. | 1 | PhP | NA | Pain relief 78% | Wound infection |

**Supplementary table 1**. Description of the series of cases focused in motor cortex stimulation.

CPS - central post-stroke pain syndrome; TN - trigeminal neuralgia; PPTN - peripheral posttraumatic neuropathy; SCI - spinal cord injury, BPI - brachial plexus injury; PhP -phantom pain; TS -trial stimulation; # -more than 50% pain relief; NA - not applicable (trial stimulation was not performed); * – patients without improvements in trial were excluded.

**Supplementary References**

S1. Tsubokawa, T., Katayama, Y., Yamamoto, T., Hirayama, T. & Koyama, S. Treatment of thalamic pain by chronic motor cortex stimulation. *Pacing Clin. Electrophysiol.* **14**, 131–134 (1991).

S2. Meyerson, B. A., Lindblom, U., Linderoth, B., Lind, G. & Herregodts, P. Motor cortex stimulation as treatment of trigeminal neuropathic pain. *Acta Neurochir. Suppl. (Wien)* **58**, 150–153 (1993).

S3. Ebel, H., Rust, D., Tronnier, V., Böker, D. & Kunze, S. Chronic precentral stimulation in trigeminal neuropathic pain. *Acta Neurochir. (Wien)* **138**, 1300–1306 (1996).

S4. Nguyen, J. P. *et al.* Motor cortex stimulation in the treatment of central and neuropathic pain. *Arch. Med. Res.* **31**, 263–265 (2000).

S5. Carroll, D. *et al.* Motor cortex stimulation for chronic neuropathic pain: a preliminary study of 10 cases. *Pain* **84**, 431–437 (2000).

S6. Roux, F. E., Ibarrola, D., Lazorthes, Y. & Berry, I. Chronic motor cortex stimulation for phantom limb pain: a functional magnetic resonance imaging study: technical case. *Neurosurgery* **48**, 681–688 (2001).

S7. Rasche, D., Ruppolt, M., Stippich, C., Unterberg, A. & Tronnier, V. M. Motor cortex stimulation for long-term relief of chronic neuropathic pain: a 10 year experience. *Pain* **121**, 43–52 (2006).

S8. Delavallée, M., Abu-Serieh, B., De Tourchaninoff, M. & Raftopoulos, C. Subdural motor cortex stimulation for central and peripheral neuropathic pain: A long-term follow-up study in a series of eight patients. *Neurosurgery* **63**, 101–105 (2008).

S9. Velasco, F. *et al.* Efficacy of motor cortex stimulation in the treatment of neuropathic pain: a randomized double-blind trial. *J. Neurosurg.* **108**, 698–706 (2008).

S10. Lefaucheur, J. P. *et al.* Motor cortex stimulation for the treatment of refractory peripheral neuropathic pain. *Brain* **132**, 1463–1471 (2009).

S11. Raslan, A. M., Nasseri, M., Bahgat, D., Abdu, E. & Burchiel, K. J. Motor cortex stimulation for trigeminal neuropathic or deafferentation pain: an institutional case series experience. *Stereotact. Funct. Neurosurg.* **89**, 83–88 (2011).

S12. Pereira, E. A. C., Moore, T., Moir, L. & Aziz, T. Z. Long-term motor cortex stimulation for phantom limb pain. *Br. J. Neurosurg.* **29**, 272–274 (2015).
